# So Shiho Tang Reduces Inflammation in Lipopolysaccharide-Induced RAW 264.7 Macrophages and Dextran Sodium Sulfate-Induced Colitis Mice

**DOI:** 10.3390/biom14040451

**Published:** 2024-04-07

**Authors:** Mei Tong He, Geonha Park, Do Hwi Park, Minsik Choi, Sejin Ku, Seung Hyeon Go, Yun Gyo Lee, Seok Jun Song, Chang-Wook Ahn, Young Pyo Jang, Ki Sung Kang

**Affiliations:** 1 College of Korean Medicine, Gachon University, Seongnam 13120, Republic of Korea; ellenho@gachon.ac.kr (M.T.H.); parkdo@gachon.ac.kr (D.H.P.); 2 Department of Life and Nanopharmaceutical Sciences, Graduate School, Kyung Hee University, Seoul 02447, Republic of Korea; ginapark0326@khu.ac.kr; 3 Department of Biomedical and Pharmaceutical Sciences, Graduate School, Kyung Hee University, Seoul 02447, Republic of Korea; alstlr7595@naver.com (M.C.); zbxl0910@khu.ac.kr (S.K.); 2021310688@khu.ac.kr (S.H.G.); kyo3733@naver.com (Y.G.L.); thd105@naver.com (S.J.S.); 4 Dr. Ahn’s Surgery Clinic, Osan 18144, Republic of Korea; acwe7@naver.com; 5 Department of Integrated Drug Development and Natural Products, Graduate School, Kyung Hee University, Seoul 02447, Republic of Korea

**Keywords:** So Shiho Tang, saikosaponins, ginsenoside Rb_1_, baicalin, glycyrrhizic acid, macrophages, ulcerative colitis

## Abstract

So Shiho Tang (SSHT) is a traditional herbal medicine commonly used in Asian countries. This study evaluated the anti-inflammatory effect of SSHT and the associated mechanism using lipopolysaccharide (LPS)-stimulated RAW 264.7 macrophages and murine dextran sodium sulfate (DSS)-induced ulcerative colitis models. Pre-treatment of RAW 264.7 macrophages with SSHT significantly reduced LPS-induced inflammation by decreasing nitrite production and regulating the mitogen-activated protein kinase pathway. Meanwhile, in mice, DSS-induced colitis symptoms, including colon shortening and body weight loss, were attenuated by SSHT. Moreover, representative compounds of SSHT, including glycyrrhizic acid, ginsenoside Rb_1_, baicalin, saikosaponin A, and saikosaponin B2, were quantified, and their effects on nitrite production were measured. A potential anti-inflammatory effect was detected in LPS-induced RAW 264.7 cells. Our findings suggest that SSHT is a promising anti-inflammatory agent. Its representative components, including saikosaponin B2, ginsenoside Rb_1_, and baicalin, may represent the key active compounds responsible for eliciting the anti-inflammatory effects and can, therefore, serve as quality control markers in SSHT preparations.

## 1. Introduction

Inflammatory responses are a major component of the immune system’s defense against harmful stimuli [1]. Ulcerative colitis (UC) is an inflammatory bowel disease (IBD) that affects the colon and rectum [2]. Patients with UC experience recurrent gastrointestinal symptoms, including abdominal pain, bloody diarrhea, rectal urgency, tenesmus, and weight loss [3]. Although the precise etiology of UC is complex and not fully defined, evidence implicates genetics, lifestyle, gut microbiota, and immune responses in its pathogenesis [4]. The current therapies for UC include steroids, immunomodulators, and surgery [5]. Moreover, preventing the occurrence of chronic inflammatory conditions may help improve UC symptoms [6].

During the inflammatory process, macrophages are activated by inflammatory stimuli, such as lipopolysaccharide (LPS), to secrete inflammatory mediators [7]. For example, sensing of LPS by toll-like receptor (TLR)-4 activates the mitogen-activated protein kinase (MAKP) pathway, resulting in nitric oxide (NO) and prostaglandin E2 secretion and subsequent expression of inducible nitric oxide synthase (iNOS) and cyclooxygenase-2 (COX-2) [8,9]. Additionally, the transcription factor nuclear factor erythroid 2-related factor 2 (NRF2) functions as a modulator of redox homeostasis [10]. Several studies have reported the role that NRF2 plays in macrophage activation by alleviating the macrophage inflammatory response by inhibiting pro-inflammatory cytokine transcription [11]. Khor et al. [12] also reported an increased expression of inflammatory markers, including iNOS and COX-2, in *Nrf2*-deficient mice, which increased their susceptibility to dextran sulfate sodium (DSS)-induced colitis. Therefore, exploring these pathways to suppress the inflammatory response may be a potential approach for protecting against inflammation-related diseases.

Traditional herbal medicines are widely used for the prevention and treatment of UC, symptom remission, and inflammatory inhibition [13]. So Shiho Tang (SSHT, Xiao Chai Hu Tang in Chinese and Sho-saiko-to in Japanese) is a combined formula of seven herbs: *Bupleurum* root, *Pinellia* tuber, jujube fruit, ginseng root, glycyrrhiza root, ginger rhizome, and *Scutellaria* root [14]. SSHT is traditionally used to treat chronic liver diseases, infectious diseases, and gastrointestinal disorders [14]. However, the mechanism or mechanisms underlying the protective effects of SSHT against inflammation-related UC remain unclear. This study investigated the beneficial effects of SSHT on the inflammatory response in LPS-induced RAW 264.7 macrophages and colitis in DSS-induced mice. Additionally, five compounds from SSHT were selected as representative components and subjected to chromatography and quantitation.

## 2. Materials and Methods

### 2.1. Materials and Reagents

For sample extraction, ethanol, methanol, and ethyl acetate (extra-pure-grade) were purchased from Duksan Pure Chemicals Co. (Ansan, Republic of Korea). For high-performance liquid chromatography (HPLC) quantification, HPLC-grade acetonitrile, methanol, and water were purchased from Thermo Fisher Scientific (Waltham, MA, USA). Formic acid (99%, HPLC-grade) was obtained from Wako Pure Chemical Industries Ltd. (Osaka, Japan), and acetic acid (99%) and LPS (from *Escherichia coli* O111:B4, L4391) were provided by Sigma-Aldrich (St. Louis, MO, USA). Glycyrrhizic acid (CAS No. 1405-86-3, CFN99151, CFS202002, purity 99.5%), ginsenoside Rb_1_ (CAS No. 41753-43-9, CFN99964, CFS202101, purity 98.9%), baicalin (CAS No. 21967-47-9, CFN99111, CFS202003, purity 98.0%), saikosaponin A (CAS no. 20736-09-8, CFN99987, CFS202102, purity 99.4%), and saikosaponin B2 (CAS No. 58316-41-9, CFN99126, CFS202102, purity 99.7%) were supplied by Chemfaces (Wuhan, China). Antibodies against NOS2 (iNOS), COX-2, p38, phosphorylated-p38 (pp38), ERK, phosphorylated-ERK (pERK), JNK, phosphorylated-JNK (pJNK), NRF2, β-actin, anti-rabbit and anti-mouse IgG HRP-linked antibodies were purchased from Cell Signaling Technology (Danvers, MA, USA) and Santa Cruz Biotechnology (Santa Cruz, CA, USA).

### 2.2. Solution Preparation

A 1000 μg/mL glycyrrhizic acid stock solution was prepared using 70% methanol, passed through a 0.2 μm polyvinylidene fluoride (PVDF) syringe filter (Whatman International Ltd. Maidstone, Kent, UK), and serially diluted to obtain 15.625, 31.25, 62.5, 250, and 500 μg/mL standard solutions. An 800 μg/mL ginsenoside Rb_1_ stock solution was prepared using water, passed through a 0.2 μm PVDF syringe filter, and diluted serially to obtain 50, 100, 200, 400, and 800 μg/mL standard solutions. A 1000 μg/mL baicalin stock solution was prepared using 70% ethanol, passed through a 0.2 μm PVDF syringe filter, and serially diluted to obtain 15.625, 31.25, 125, 500, and 1000 μg/mL standard solutions. Saikosaponin A and B2 stock solutions (1000 μg/mL) were prepared using methanol, passed through a 0.2 μm PVDF syringe filter, and serially diluted to obtain 7.8125, 15.625, 62.5, 125, and 250 μg/mL standard solutions for saikosaponin A and 15.625, 31.25, 62.5, 250, and 1000 μg/mL for saikosaponin B2. Three standard (calibration) curves were prepared from the diluted solutions at five points (*n* = 6/point). 

### 2.3. Sample Preparation

SSHT dry extract (HC-DE-2101; Hanpoong Pharm & Foods, Wanju, Republic of Korea) comprises the root of *Bupleurum falcatum* L. (2.33 g), tuber of *Pinellia ternata* Breitenbach (1.67 g), fresh rhizome of *Zingiber officinale* Roscoe (1.33 g), root of *Scutellaria baicalensis* Georgi (1.00 g), fruit of *Zizyphus jujuba* Miller var. inermis Rehder (1.00 g), root of *Panax ginseng* C. A. Meyer (1.00 g), and root and rhizome of *Glycyrrhiza uralensis* Fischer (0.67 g). 

To quantify glycyrrhizic acid in *G. uralensis*, one dose (1.31 g) of SSHT powder was extracted with 50 mL of 70% methanol, refluxed for 2 h, and filtered; 70% methanol was added to a final volume of 100 mL. To extract *P. ginseng* ginsenoside Rb_1_, two doses (2.62 g) of SSHT powder were treated with 100 mL of 70% methanol, refluxed for 30 min, filtered, and concentrated to dryness. The residue was dissolved in 10 mL of water and used as the sample solution. For baicalin in *S. baicalensis*, one dose (1.31 g) of SSHT powder was treated with 100 mL of 70% ethanol, refluxed for 1 h, and filtered; 70% ethanol was again added to a final volume of 100 mL. For saikosaponin A and B2 in *B. falcatum*, two doses (2.62 g) of SSHT powder were treated with 50 mL of 80% methanol, refluxed for 2 h, filtered, and concentrated to dryness. The residue was dissolved in water (50 mL) and extracted twice with ethyl acetate (50 mL). The ethyl acetate fraction was concentrated to dryness and dissolved in methanol (2 mL). All sample solutions were passed through a 0.2 μm PVDF syringe filter before being injected for HPLC analysis.

### 2.4. HPLC Analysis Conditions

Four different analytical methods were developed, validated, and applied for the quantitative analysis of the five representative compounds extracted from SSHT. The details of each analysis method, equipment, and column used are summarized in Table 1.

### 2.5. Validation of HPLC Quantification Method

The HPLC assay methods developed for use in SSHT quality assurance were authenticated by evaluating and verifying numerous factors, including specificity, linearity, detection and quantitation thresholds, accuracy, precision, and robustness. These analyses complied with ICH Guideline Q2(R2) [15].

#### 2.5.1. Specificity

The retention times and UV spectra of the standard compounds and test samples were compared to ensure that the target compounds were accurately identified and distinguished from other components in the sample. For specificity data, HPLC chromatograms and UV spectra were generated for standard compounds and sample solutions. Furthermore, accuracy studies were conducted to validate the specificity of the analytical method. This involved analyzing known standard compounds with well-defined properties and concentrations. The accuracy and reliability of the analytical method were assessed by comparing the obtained results with the expected values.

#### 2.5.2. Working Range (Linearity, Detection Limit [DL], and Quantitation Limit [QL])

The linearity between analyte concentrations and responses was assessed throughout the working range of the analytical procedure. This involved plotting the signals obtained as a function of the analyte concentration or content. The test results were evaluated by generating a regression line using the least squares method. A minimum of five concentrations were selected and appropriately distributed across the desired range to establish linearity. Three replicate injections were administered at each concentration, resulting in three linear lines. This approach ensured the reliability and accuracy of the linearity assessment.

The DL is the lowest concentration of analyte that can be reliably detected; the QL is the lowest concentration that can be accurately quantified within the specified limits of precision and accuracy. The DL and QL were estimated using the approach described in the “Based on the Standard Deviation of a Linear Response and a Slope” ICH Q2(R2) Guideline [15], according to Equations (1) and (2):

(1) $$DL=\frac{3.3\sigma }{S}$$

(2) $$QL=\frac{10\sigma }{S}$$

where the slope of the calibration curve, S, was estimated from the regression line of the analyte, whereas the σ (the standard deviation of the response) estimate was obtained from the standard deviation of the y-intercepts of the regression lines.

#### 2.5.3. Accuracy

Accuracy was assessed throughout the reportable range of the analytical procedure and was commonly demonstrated by comparing the measured results with an expected or known value. To evaluate the accuracy of the analytical procedure, known concentrations of glycyrrhizic acid, ginsenoside Rb_1_, baicalin, saikosaponin A, and saikosaponin B2 standards were spiked into the SSHT sample solutions. Before the addition of these five chemical standards, the contents of glycyrrhizic acid, ginsenoside Rb_1_, baicalin, saikosaponin A, and saikosaponin B2 in the sample solutions were determined. The accuracy of the analytical procedure was determined and verified by comparing the measured and expected values.

#### 2.5.4. Precision (Repeatability, Intermediate Precision, and Reproducibility)

Repeatability can be assessed using two different procedures. A minimum of nine determinations should be performed, covering the reportable range of analytical procedures. This typically involves analyzing samples at different concentrations, with three concentrations selected and each tested in triplicate. Alternatively, in this study, a minimum of six measurements were conducted at a test concentration of 100%. This ensured the procedure consistently produced reliable and precise results across the reported range at the desired test concentration. 

The same experiment was conducted on different days in the same laboratory to assess intermediate precision. This evaluated the variability in results due to factors such as analysts, equipment, or environmental conditions, which may vary daily. 

Reproducibility was evaluated through an inter-laboratory trial, wherein the developed analytical procedures were performed by the Korea Pharmaceutical Test and Research. This assessed the performance of methods when implemented in an external laboratory setting.

#### 2.5.5. Robustness

Robustness testing was conducted to demonstrate the reliability and resilience of the analytical procedure under intentional variations or perturbations in the parameters. Therefore, three columns with similar specifications were tested under identical analytical conditions. By subjecting the method to robustness testing with different brand columns but the same specifications, we were able to assess the method’s ability to consistently deliver accurate and reliable results despite variations in column sources or manufacturers.

### 2.6. Cell Culture

RAW 264.7 macrophages were purchased from the American Type Culture Collection (Manassas, VA, USA). They were cultured at 37 °C in a humidified atmosphere containing 5% CO_2_ in Dulbecco’s Modified Eagle Medium (Corning, Mediatech Inc., Manassas, VA, USA) supplemented with 10% fetal bovine serum (FBS, Atlas Biologicals, Fort Collins, CO, USA) and 1% penicillin/streptomycin (Gibco BRL, Carlsbad, MD, USA). The in vitro groups comprised the normal group, i.e., a vehicle group that did not receive treatment; the LPS group, i.e., a control group treated with only LPS; and the SSHT groups treated with LPS and various concentrations of SSHT.

### 2.7. Cell Viability Assay

RAW 264.7 cells were seeded on a 96-well plate at a density of 1 × 10^5^ cells per well and incubated for 24 h. Subsequently, the cells were treated with various concentrations of SSHT (50, 100, 250, 500, 750, or 1000 µg/mL) or the individual compounds (saikosaponin A, saikosaponin B2, ginsenoside Rb_1_, baicalin, and glycyrrhizic acid) at concentrations of 5, 25, and 50 µM for 2 h before being treated with 1 µg/mL LPS. After 24 h of incubation, cell viability was measured using an Ez-Cytox kit (DoGenBio, Seoul, Republic of Korea).

### 2.8. Griess Assay

Cells were seeded in a 96-well plate and incubated for 24 h. The cells were pre-treated with SSHT or the compounds (saikosaponin A, saikosaponin B2, ginsenoside Rb_1_, baicalin, and glycyrrhizic acid) at various concentrations and subsequently treated with LPS. After a 24 h incubation, 100 µL of the supernatant was mixed with 100 µL of Griess reagent (0.1% naphthylethylenediamide and 1% sulfanilamide in 5% phosphoric acid). Cells treated with dexamethasone (Dex, 10 µM) served as a positive control. The absorbance at 540 nm was measured using a microplate reader (PowerWave XS; Bio-Tek Instruments, Winooski, VT, USA).

### 2.9. Western Blot Analysis

Cells were seeded in a 6-well plate at a density of 1 × 10^6^ cells/mL for 24 h. Subsequently, they were treated with SSHT (100 and 250 µg/mL) before LPS treatment. The cells were then washed with DPBS and lysed using a lysis buffer (radio-immunoprecipitation assay buffer supplemented with a protease inhibitor cocktail). The disrupted cells were centrifuged at 13,400× *g* at 4 °C for 20 min. The supernatant was collected, and the protein concentration was measured using a BCA protein assay kit (Thermo Fisher Scientific). Thirty-microgram samples of protein were loaded onto 8% and 10% SDS polyacrylamide gels and transferred to polyvinylidene difluoride membranes. The membranes were blocked for non-specific binding with 5% skim milk for 1 h. Subsequently, they were washed with TBST and incubated with primary antibodies (p38, pp38, JNK, pJNK, ERK, pERK, NRF-2, NOS2, COX-2, and β-actin) at 4 °C overnight. The membranes were washed with TBST and incubated with secondary antibodies (anti-rabbit or anti-mouse) for 1 h at room temperature. β-Actin served as the loading control. Each treatment condition was run in triplicate lanes, and protein bands were detected using a FUSION Solo chemiluminescent detection system (Vilber Lourmat Deutschland GmbH, Eberhardzell, Germany).

### 2.10. Animals Experiment

All experimental procedures involving animals were reviewed and approved by the Institutional Animal Care and Use Committee of Gachon University (approval number: GU1-2022-IA0008-00). Male BALB/c mice, aged 5 weeks and weighing 20–21 g, were purchased from DBL (Chungcheongbuk-do, Republic of Korea). Mice were housed in cages, fed standard laboratory chow under 12 h light/dark cycles, and acclimatized for 1 week before the experiment. Thereafter, the mice were randomly divided into four groups (*n* = 5 per group): (1) control group (normal), (2) DSS group (control), (3) DSS + SSHT 250 mg/kg/day, and (4) DSS + SSHT 500 mg/kg/day. Mice were administered 2.5% DSS in sterilized drinking water for 5 days to induce colitis [16,17]. The control mice received only drinking water. Mice were orally administered SSHT or saline solution for 10 days, starting the first day of DSS treatment. Body weight and water intake were recorded daily. Mice were sacrificed after the final oral administration of SSHT on Day 10. The colons were collected for further analysis. 

### 2.11. General Assessment of Colitis

The disease activity index (DAI) was used to evaluate the severity of colonic inflammation. DAI was calculated based on body weight change, fecal consistency, and fecal blood [18]. To measure the length of the colon, it was placed on a flat surface and measured using a ruler.

### 2.12. Statistical Analysis

Results were represented as the mean ± standard deviation (SD). All data were analyzed using Statistical Package for Social Sciences version 26.0 (SPSS Inc., Chicago, IL, USA). Comparisons between groups were performed using one-way analysis of variance with post hoc Tukey’s analysis. *p* < 0.05 was considered statistically significant. 

## 3. Results

### 3.1. Chromatography and Quantitation of the Five Compounds in SSHT

**Figure 1:**
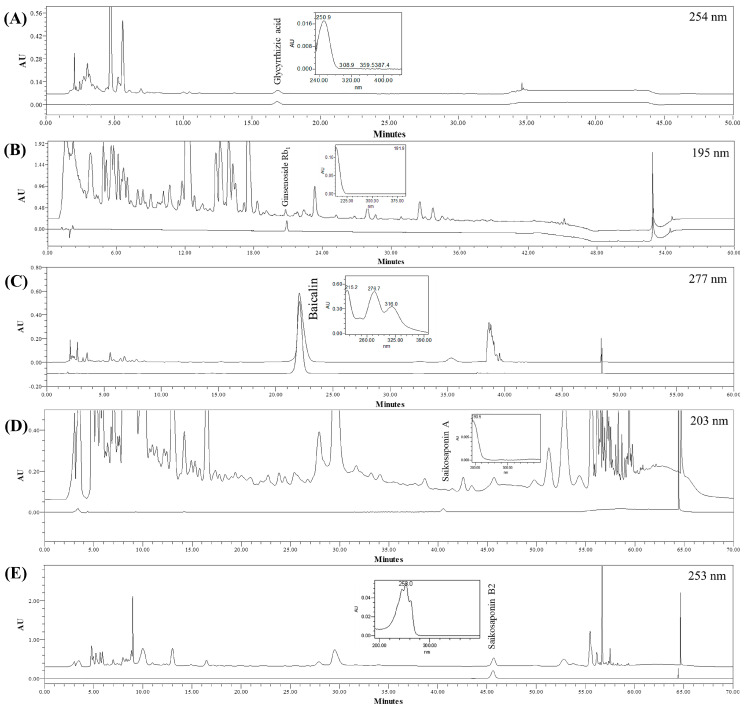
HPLC profiles of SSHT. Glycyrrhizic acid ((**A**), 254 nm), ginsenoside Rb_1_ ((**B**), 195 nm), baicalin ((**C**), 277 nm), saikosaponin A ((**D**), 203 nm), and saikosaponin B2 ((**E**), 253 nm). The upper line represents the SSHT sample, and the lower line represents the standard solution. The concentration of each marker is as follows: glycyrrhizic acid, 62.5 µg/mL; ginsenoside Rb_1_ and baicalin, 500 µg/mL; and saikosaponin A and B2, 250 µg/mL.

Five compounds, glycyrrhizic acid from *Glycyrrhizae* Radix, ginsenoside Rb_1_ from *Ginseng* Radix, baicalin from *Scutellariae* Radix, and saikosaponin A and B2 from *Bupleuri* Radix, were chosen as markers of SSHT dry extract. The specificity of the assay methods was ascertained by juxtaposing the chromatographic profile and the data derived from the standards and the sample, considering factors such as retention time and UV spectra (Figure 1). 

The developed assay methods were assessed based on linearity, DL, QL, accuracy, precision, and robustness for validation. The regression equation and coefficient (r^2^) showed superior linearity from 0.9990 to 0.9996 across all markers (Table 2). This was established based on the prepared calibration curves. For glycyrrhizic acid, ginsenoside Rb_1_, baicalin, saikosaponin A, and saikosaponin B2, the DL was 5.24, 4.40, 3.08, 0.73, and 3.46 µg/mL and the QL was 15.89, 13.33, 9.34, 2.21, and 10.49 µg/mL, respectively. 

The accuracy of the analytical procedure was assessed by spiking the sample solutions with known amounts of chemical standards. The mean recoveries (%) at low, medium, and high concentrations within the working range were 98.60%, 97.44%, 102.26%, 97.19%, and 117.73% for glycyrrhizic acid, ginsenoside Rb_1_, baicalin, saikosaponin A, and saikosaponin B2, respectively. By performing this accuracy evaluation, the reliability and precision of the analytical procedure for determining the concentrations of these compounds in SSHT samples were verified. 

Precision was validated using the relative standard deviation (RSD, %) of repeatability, intermediate precision, and reproducibility. All RSD values for the repeatability were below 2.00%, excluding the 100% concentration repeatability of ginsenoside Rb_1_, which was 2.62%, indicating appropriate precision validation results. For intermediate precision and reproducibility, all RSD values remained below 2.49% and 3.92%, respectively. 

The robustness was verified by assessing the system suitability of the analyte peak in the sample solution (%RSD of area, capacity factor, symmetry factor, and resolution) when an identical analysis method was performed using three different brands of columns with similar specifications. The comprehensive validation results are presented in the Supplementary Materials, specifically in Tables S1–S5. 

The glycyrrhizic acid and baicalin contents in SSHT adhered to the Korean Herbal Pharmacopoeia (KHP) criteria. The contents of additional indicator substances, such as ginsenoside Rb_1_, saikosaponin A, and saikosaponin B2, are shown in Table 3.

### 3.2. Effects of Saikosaponin A, Saikosaponin B2, Ginsenoside Rb1, Baicalin, and Glycyrrhizic Acid on Nitrite Production in LPS-Stimulated RAW 264.7 Cells

**Figure 2:**
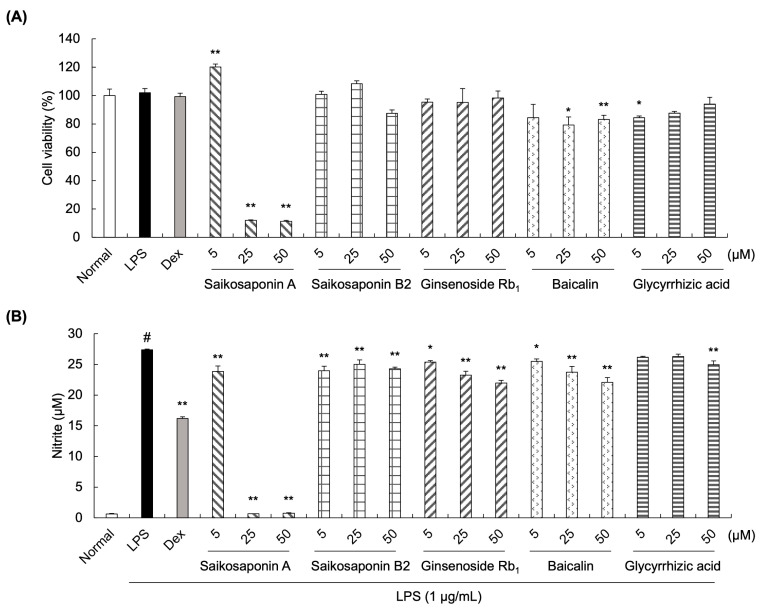
Effect of SSHT components on cell viability and nitrite production in lipopolysaccharide (LPS)-stimulated RAW 264.7 cells. (**A**) Viability of LPS-stimulated RAW 264.7 cells following treatment with saikosaponin A or B2, ginsenoside Rb_1_, baicalin, or glycyrrhizic acid. (**B**) Nitrite levels in LPS-stimulated RAW 264.7 cells treated with saikosaponin A or B2, ginsenoside Rb_1_, baicalin, or glycyrrhizic acid. Dexamethasone (Dex, 10 µM) was the positive control. Data are presented as the mean ± standard deviation (SD; *n* = 3). ^#^ *p* < 0.05 vs. normal group. * *p* < 0.05, ** *p* < 0.001 vs. LPS group.

The Griess assay was conducted to measure the potential anti-inflammatory effect of the SSHT components. Cell viability was first detected to evaluate the cytotoxic effects of saikosaponin A, saikosaponin B2, ginsenoside Rb_1_, baicalin, and glycyrrhizic acid on RAW 264.7 cells (Figure 2A). Although the viability of cells treated with saikosaponin B2, ginsenoside Rb_1_, baicalin, or glycyrrhizic acid was slightly affected, it was maintained over 80%, demonstrating that these components at concentrations of 5, 25, or 50 µM could be used in subsequent experiments. However, the cell viability of saikosaponin A at 25 and 50 µM decreased to 11%, demonstrating strong cytotoxic effects on LPS-induced RAW 264.7 cells. 

Furthermore, nitrite production was evaluated (Figure 2B). LPS treatment at 1 µg/mL significantly increased the nitrite levels compared with normal untreated cells, whereas treatment with ginsenoside Rb1 and baicalin significantly decreased the nitrite levels in a concentration-dependent manner (5, 25, and 50 µM). Similar results were observed following treatment with saikosaponin B2 at all concentrations (5, 25, and 50 µM). In contrast, the nitrite levels in cells treated with 5 or 25 µM glycyrrhizic acid did not differ significantly from those of LPS-treated cells.

### 3.3. Effect of SSHT Extract on Nitrite Production and iNOS and COX-2 Expression in LPS-Stimulated RAW 264.7 Cells

**Figure 3:**
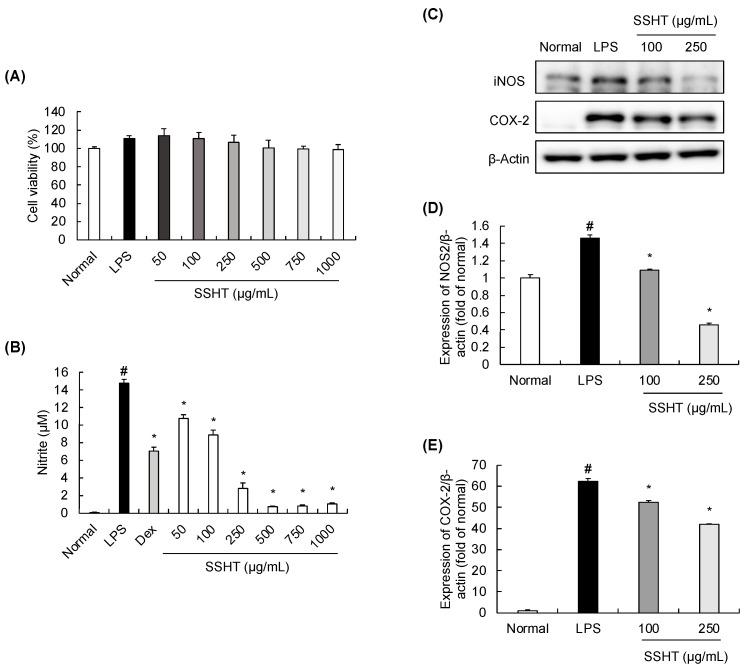
Effect of SSHT on nitrite production and inducible nitric oxide synthase (iNOS) and cyclooxygenase-2 (COX-2) abundance in LPS-stimulated RAW 264.7 cells. (**A**) Viability of RAW 264.7 cells following treatment with SSHT (50–1000 µg/mL). (**B**) Nitrite production by RAW 264.7 cells following treatment with LPS or SSHT. Dex (10 µM) was the positive control. (**C**–**E**) Abundance of iNOS and COX-2 in LPS-induced RAW 264.7 cells treated with SSHT (100 and 250 µg/mL). Data are presented as the mean ± SD (*n* = 3). ^#^ *p* < 0.05 vs. normal group. * *p* < 0.05 vs. LPS group. Original blot images can be found in Supplementary File S1.

A cell viability assay was performed to assess the cytotoxicity effect of SSHT on RAW 264.7 cells. SSHT at various concentrations (50, 100, 250, 500, 750, and 1000 µg/mL) had no significant effects on cell viability (Figure 3A). Next, we evaluated the inhibitory effect of SSHT on nitrite production (Figure 3B). Compared with the normal group, 1 µg/mL of LPS significantly increased nitrite production, which was inhibited by treatment with SSHT or Dex. In addition, the abundance of inflammatory mediators was assessed through Western blotting. LPS significantly upregulated the abundance of iNOS and COX-2 in RAW 264.7 cells, and this effect was inhibited by SSHT treatment in a concentration-dependent manner (Figure 3C–E).

### 3.4. Effect of SSHT on Modulating MAPK Signaling Pathway in LPS-Stimulated RAW 264.7 Cells

**Figure 4:**
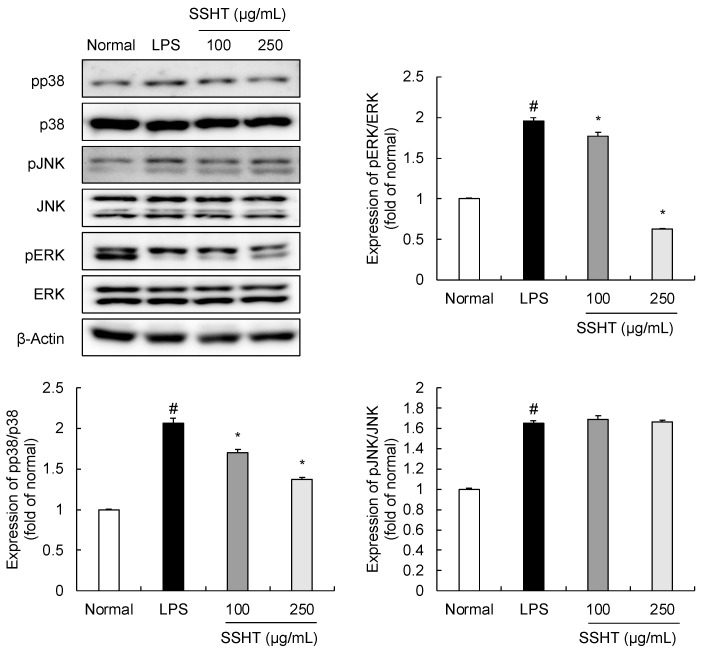
Effect of SSHT on p38, JNK, and ERK phosphorylation and total protein levels in LPS-stimulated RAW 264.7 cells. Cells stimulated with or without LPS were treated with or without SSHT (100 and 250 µg/mL). Data are presented as the mean ± SD (*n* = 3). ^#^ *p* < 0.05 vs. normal group. * *p* < 0.05 vs. LPS group. Original blot images can be found in Supplementary File S1.

The MAPK (JNK, ERK, and p38) signaling pathway has important roles in inflammatory responses, including inflammation-related diseases [19]. LPS treatment (1 µg/mL) markedly increased the phosphorylation of p38, ERK, and JNK (Figure 4). However, SSHT treatment suppressed the LPS-stimulated increase in p38 and ERK phosphorylation in a concentration-dependent manner, with no impact on total protein. However, the effect of SSHT on JNK phosphorylation did not differ significantly from the LPS control group.

### 3.5. Effect of SSHT on NRF2 Expression in LPS-Stimulated RAW 264.7 Cells

**Figure 5:**
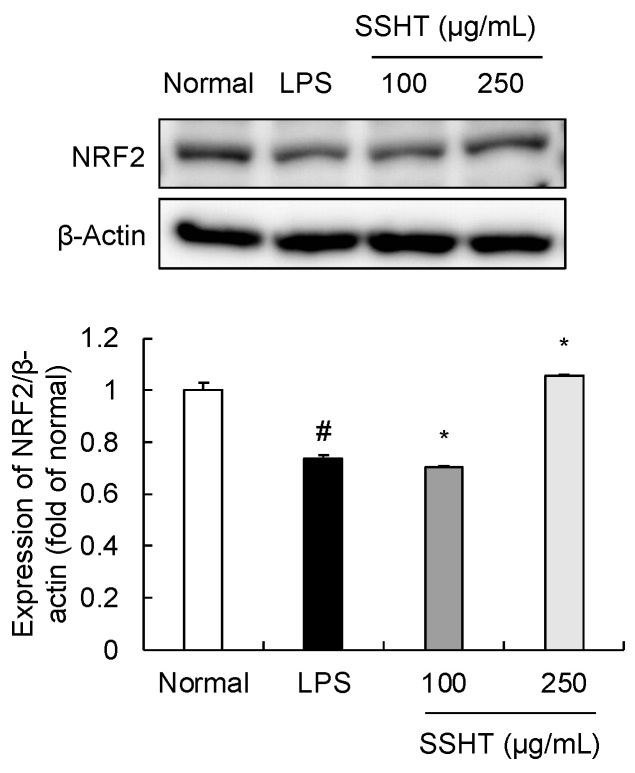
Effect of SSHT on nuclear factor erythroid 2-related factor 2 (NRF2) protein expression in LPS-stimulated RAW 264.7 cells. SSHT treatment (250 µg/mL) reversed NRF2 protein expression. Data are presented as the mean ± SD (*n* = 3). ^#^ *p* < 0.05 vs. normal group. * *p* < 0.05 vs. LPS group. Original blot images can be found in Supplementary File S1.

The transcription factor NRF2 functions as a modulator of cellular and organismal defenses against endogenous and exogenous stressors, eliciting an anti-inflammatory effect [20,21]. LPS-stimulated RAW 264.7 cells exhibited decreased NRF2 protein expression. Notably, 250 µg/mL SSHT treatment reversed this LPS-mediated decrease in NRF2 (Figure 5).

### 3.6. Effect of SSHT in DSS-Induced Colitis Mice

**Figure 6:**
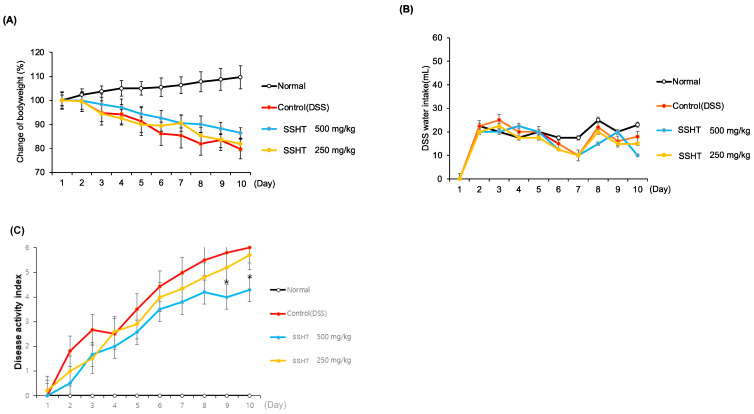
Effects of SSHT on body weight, water intake, and diarrhea status. (**A**) Body weight changes over 10 days. (**B**) Water intake. (**C**) Diarrhea status, scored using disease activity index. Values are expressed as the mean ± SD (*n* = 5). * *p* < 0.05 vs. control group.

**Figure 7:**
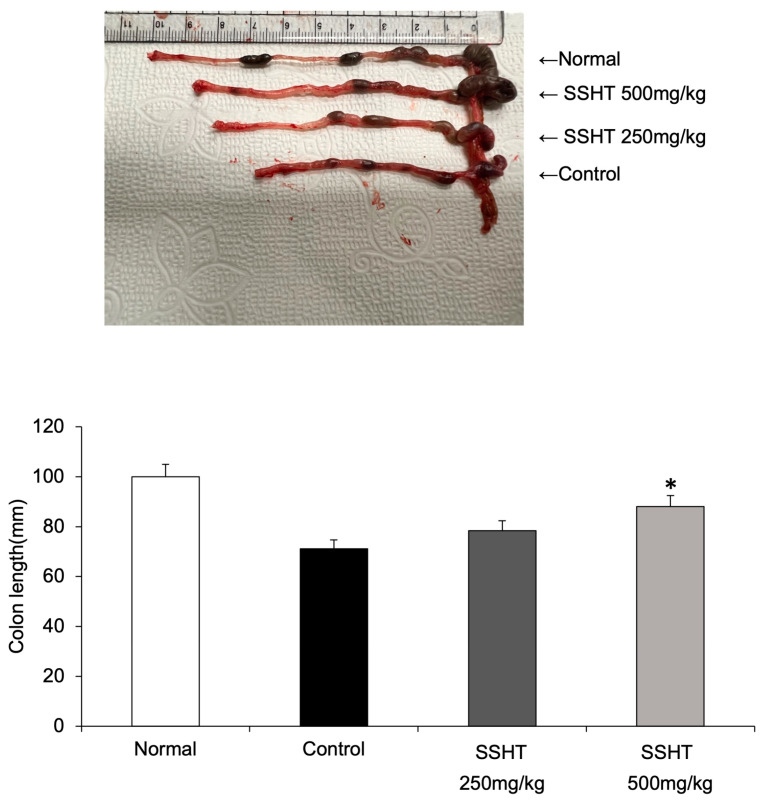
Effect of SSHT on colon length in dextran sodium sulfate (DSS)-induced colitis model mice. Colon length was measured on the last day (day 10 of DSS treatment). The colon was dissected between the ileocecal junction and the proximal rectum, close to the subpelvic passage. The colon was placed on a non-absorbable surface, and its length was measured with a ruler while ensuring that the organ was not stretched. Representatives of one out of three similar independent experiments are shown per group. * *p* < 0.05 vs. control group.

Changes in body weight, DAI, and colon length were measured to assess the protective effects of SSHT on DSS-treated mice. After 10 days of treatment, the body weight of mice in the DSS-treated group decreased in a time-dependent manner, which was ameliorated by SSHT treatment (Figure 6A). Water intake did not differ significantly among the groups (Figure 6B). Mice treated with DSS showed an increased DAI score compared with normal mice. However, SSHT at 500 mg/kg significantly decreased the DAI from day 9 (Figure 6C). Moreover, SSHT at 500 mg/kg significantly recovered colon length compared with control DSS-treated mice (Figure 7). These results indicated that SSHT has a potential protective effect in mice with DSS-induced colitis.

## 4. Discussion

Among traditional medicines, SSHT has been studied to better understand multiple therapeutic mechanisms through multiple targets and in combination therapies for various diseases [22,23]. In the present study, mice with DSS-induced colitis exhibited significant recovery of colon length and a decreased DAI following SSHT treatment. Furthermore, an anti-inflammatory effect was evidenced by reduced NO production and downregulation of inflammatory factors via the MAPK signaling pathway. Moreover, the HPLC quantification performed in this study analyzed the five selected representative compounds in SSHT. The associated methodology can be applied to achieve quality assurance during SSHT preparation.

IBD is an immune-mediated chronic relapsing inflammatory disease [24]. Although UC is a major type of IBD, its etiology is complex and not fully understood. UC is typically characterized by continuous inflammation induced by abnormal mucosal immune responses and disrupted epithelial barrier function [25,26]. Therefore, targeted anti-inflammatory agents are considered an effective option for UC management [27]. As the DSS-induced colitis model is similar to human UC, it has been established as an ideal model for studying intestinal inflammation [28]. Classical UC symptoms, including body weight loss, colon length shortening, and increased DAI, are reflected in DSS-induced colitis [29,30]. In the present study, administering DSS (2.5%) induced body weight loss, colon length shortening, and increased DAI [16,31]. Meanwhile, high-dose SSHT treatment (500 mg/kg) significantly recovered these symptoms, demonstrating suppression of the inflammatory response in DSS-induced mice. In this study, we monitored the body weight and water intake of the mice and compared the colon length of the mice as a preliminary study. Nevertheless, the present study has some limitations. For example, a detailed histopathological assessment of the colon tissues and granulocyte infiltration should be conducted in future studies. In addition, the animal dose (mg/kg) should be multiplied by the correction factor (K_m_) ratio according to the HED (human equivalent dose) [32]. Considering that 250 and 500 mg/kg of SSHT were orally administered in the current study, the HED would be 500 mg/kg × 0.081 = 40.54 mg/kg. A previous study reported that the standard SSHT tablets are 0.4 g, and 4–6 tablets are taken three times per day. Alternatively, one or two 10 g granule packets are taken three times per day [33]. Importantly, administering 250 or 500 mg/kg SSHT for 10 days to mice did not induce apparent adverse effects, toxicity, or mortality. Accordingly, the experimental doses were deemed safe by design.

Different macrophage subtypes have unique roles in intestinal inflammation and homeostasis [34,35,36]. Patients with IBD reportedly have increased proportions of pro-inflammatory macrophages (i.e., M1 phenotype) [36]. Therefore, to assess the effect of SSHT on UC-related inflammatory responses, the corresponding mechanism was explored in vitro in LPS-stimulated RAW 264.7 cells. LPS-treated RAW 264.7 cells secrete large amounts of NO radicals, indicating that the LPS-induced inflammatory response in macrophages is mediated by NO production [37]. NO is a signaling molecule crucial in inflammation pathogenesis [38]. Specifically, high NO levels can induce the release of pro-inflammatory cytokines, supporting further NO generation and continuing a cyclic process that propagates the inflammatory process [39]. The development of many inflammatory diseases, including UC, is accompanied by the activation and overexpression of iNOS and COX-2. Accordingly, their inhibitors could serve as therapeutic agents in inflammatory diseases [39,40,41,42]. Our findings revealed that SSHT treatment reduced NO levels, with a dramatic decrease at higher concentrations. However, a dose-dependent effect was not observed at a dose of 500 µg/mL onward. Notably, SSHT also suppressed iNOS and COX-2 expression in LPS-stimulated macrophages.

MAPK signaling pathways, including p38, JNK, and ERK, can be activated by macrophage stimulation to increase iNOS and COX-2 expression, which play important roles in inflammation [43,44,45]. The inactivation of the MAPK signaling pathway reduces the synthesis of pro-inflammatory cytokines, suggesting it as a potential target for anti-inflammation [46]. LPS induces inflammatory responses by activating the MAPK pathway in RAW 264.7 macrophages [47]. Consistent with the results of the previous studies, LPS stimulation activated the MAPK pathway by upregulating the expression of phosphorylated p38, JNK, and ERK in this study. Conversely, pp38 and pERK were inhibited by SSHT in a concentration-dependent manner. Notably, pJNK was not affected by SSHT. These findings suggest that the anti-inflammatory activity of SSHT may be related to the MAPK signaling pathway by inhibiting LPS-induced phosphorylation of p38 and ERK.

NRF2 acts as a promoter to regulate the expression of cytoprotective genes against oxidative stress and inflammatory responses [48,49]. Under stress, NRF2 can be liberated from the KEAP–NRF2 complex and facilitate its subsequent translocation [50]. LPS, a component of bacterial cell walls, activates the TLR-4 on host cells to initiate oxidative stress and inflammatory responses [51]. Reactive oxygen and nitrogen species contribute to LPS-triggered macrophage activation by regulating nuclear transcription factors, including NRF2 [52]. In this study, LPS treatment increased NO generation and iNOS protein expression, demonstrating that LPS induced oxidative stress and the inflammatory response. Moreover, LPS decreased NRF2 expression in RAW 264.7 cells, indicating that LPS may inhibit NRF2 ubiquitination and proteasomal degradation by activating oxidative stress in macrophages. Notably, SSHT treatment suppressed NO production and increased NRF2 expression in LPS-stimulated RAW 264.7 cells, demonstrating that SSHT may exert an anti-inflammatory effect by activating NRF2 expression. However, the relationship between LPS stimulation and NRF2 activation, as well as the underlying mechanisms by which SSHT promotes KEAP1 degradation and/or prevents NRF2 ubiquitination and proteasomal degradation, remain unclear and require further investigation. NRF2 can attenuate intestinal oxidative stress and inflammatory factors by polarizing macrophages toward the M2 anti-inflammatory phenotype [53]. In the current study, high concentrations of SSHT significantly upregulated NRF2 expression in LPS-stimulated RAW 264.7 cells. Therefore, SSHT might upregulate NRF2 expression to exert an anti-inflammatory effect, direct macrophage polarization, and normalize immune processes in the colonic mucosa and submucosa [53].

Activation of NRF2 by kinases such as p38, ERK, and JNK is assumed to promote its release from KEAP1 and subsequent nuclear translocation [54]. Therefore, considering the close relationship between KEAP1/NRF2 and HO-1, additional analyses are required to elucidate the effects of SSHT on members of the KEAP1/NRF2/ARE signaling pathway and its downstream genes.

The KHP mandates that, per 1.0 g of dry matter, SSHT dry extract should contain at least 1.6 mg of glycyrrhizic acid from *Glycyrrhizae* Radix and a minimum of 11.9 mg of baicalin from *Scutellariae* Radix, thus establishing the quality standards for SSHT preparation. In this study, 1 g of SSHT dry extract contained 399.6, 133.3, 107.1, 35.2, and 16.6 mg of baicalin, glycyrrhizic acid, saikosaponin B2, ginsenoside Rb_1_, and saikosaponin A, respectively. Saikosaponin A induced cytotoxic effects at 25 and 100 µM. Similar findings showing cytotoxic effects of saikosaponin A on LPS-induced RAW 264.7 cells at concentrations of 12.5–100 µM were also reported in a previous study [55]. In contrast, treatment of LPS-induced RAW 264.7 cells with saikosaponin B2 (5–50 µM) induced a mild inhibitory effect on NO production, which was consistent with a previous study, indicating that NO production may be effectively suppressed by saikosaponin B2 at higher doses [56]. Moreover, Uto et al. [57] indicated that glycyrrhizic acid alone cannot suppress the LPS-induced increase in NO production but could synergistically suppress NO production with the other constituents of licorice. Meanwhile, Park et al. [58] reported that ginsenoside Rb1 exhibited NO inhibitory activity with an IC_50_ value > 0.05 mM, similar to the results of the present study. Moreover, Kuo et al. [59] indicated that 0.1 µg/mL LPS induced NO generation, which was dose-dependently inhibited by baicalin (10–50 µM). Similarly, we observed that baicalin elicited NO inhibitory activity in a dose-dependent manner. Therefore, higher doses of the single compounds from SSHT may be required to inhibit NO production. Meanwhile, SSHT showed a significant inhibitory effect on NO production, confirming that the synergistic and additive effects of medicinal herbs and herbal extracts can enhance therapeutic effects [60].

Natural flavonoids, including baicalin, can effectively treat inflammatory disorders [61]. Baicalin, a major bioactive component of *Scutellariae* Radix, is traditionally used to treat diarrhea, high blood pressure, inflammation, and respiratory infections [62]. Wang et al. [63] reported that baicalin is distributed throughout the intestinal tract and exerts protective effects against IBD by repressing inflammatory responses. In vitro and in vivo, baicalin reduces colon injury in colitis animal models and regulates pro-inflammatory mediator proteins in LPS-induced RAW 264.7 cells [64,65]. Furthermore, saponins from many popular herbal medicines, such as ginseng and bupleurum, exert strong anti-inflammatory effects on intestinal inflammation-related digestive diseases [66]. Meanwhile, glycyrrhizic acid is a triterpenoid saponin isolated from *Glycyrrhizae* Radix that has been used as a drug carrier and supplement to other drugs in traditional Chinese medicine to reduce toxicity and improve efficacy [67,68,69]. *Glycyrrhizae* Radix extract or its components exert excellent anti-inflammatory properties, accounting for their use in relieving cough and alleviating pain [70]. Moreover, glycyrrhizic acid nanoparticles reduce the levels of inflammatory mediators increased by LPS in RAW 264.7 cells [71]. Mechanistically, glycyrrhizic acid exerts anti-inflammatory responses by suppressing p38/MAPK and nuclear factor-ĸB (NF-ĸB) p65 signaling in colitis models [72]. Similarly, saikosaponins—the main bioactive components of *Bupleuri* Radix—are widely used to treat inflammatory diseases [73]; saikosaponin B2 enhances anti-inflammatory activity by blocking the NF-ĸB signaling pathway in LPS-induced macrophages [56]. Additionally, saikosaponins alleviate DSS-induced colitis by regulating the NRF2/heme oxygenase-1 pathway [74]. Over 80 saponins have been isolated from *P. ginseng* (also known as Korean ginseng) [75]. Ginsenoside Rb_1_ exerts neuroprotective, anti-obesity, and anti-depression effects by suppressing different inflammatory pathways, including oxidative stress, the NF-ĸB/MAPK pathway, the AKT pathway, and amyloidogenic processes [76,77,78]. Based on these studies, saikosaponins, baicalin, glycyrrhizic acid, and ginsenoside Rb_1_ may represent marker compounds in SSHT with important anti-inflammatory properties. These compounds could be isolated to investigate their efficacy against inflammation-related diseases.

**Figure 8:**
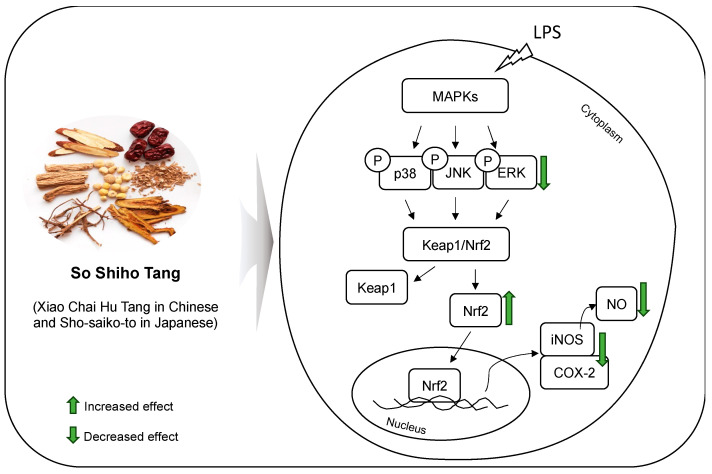
Effect of SSHT on LPS-stimulated RAW 264.7 macrophages.

In conclusion, the findings of this study demonstrate the anti-inflammatory effects of SSHT in DSS-induced colitis mice and LPS-stimulated RAW 264.7 macrophages (Figure 8). Quality assessment was performed on key anti-inflammatory components of SSHT, namely saikosaponin A and B2, baicalin, glycyrrhizic acid, and ginsenoside Rb_1_, all of which inhibited nitrite production at high concentrations, with the exception of saikosaponin A. Collectively, these results provide a theoretical foundation for future studies on SSHT and its bioactive compounds.

## Figures and Tables

**Table 1:** Quantitative analysis methods for glycyrrhizic acid, ginsenoside Rb_1_, baicalin, saikosaponin A and B2 in So Shiho Tang (SSHT).

	Glycyrrhizic Acid			Ginsenoside Rb_1_			Baicalin			Saikosaponin A and B2		
HPLC Instrument	Waters 2695 Separation Module			Waters e2695 Separation Module			Waters e2695 Separation Module			Waters e2695 Separation Module		
Detector	Waters 2996 PDA			Waters 2998 PDA			Waters 2998 PDA			Waters 2998 PDA		
Column	Phenomenex Gemini^®^ C18 110 Å column (5 μm, 4.6 × 250 mm)			YMC-Pack Pro C18 LC column (5 μm, 4.6 × 150 mm)			Luna^®^ C18(2) 100 Å column (5 μm, 4.6 × 250 mm)			Phenomenex Gemini^®^ C18 110 Å column (5 μm, 4.6 × 250 mm)		
Column Temp.	25 °C			25 °C			25 °C			25 °C		
Sample Temp.	20 °C			25 °C			20 °C			25 °C		
Detection	254 nm			195 nm			277 nm			203 nm (saikosaponin A) 253 nm (saikosaponin B2)		
Flow Rate	1.0 mL/min			1.0 mL/min			1.0 mL/min			0.8 mL/min		
Injection	10 μL			10 μL			10 μL			10 μL		
Mobile Phase	A: Methanol (0.5% Formic acid) B: Water (0.5% Formic acid)			A: Acetonitrile (0.1% Formic acid) B: Water (0.1% Formic acid)			A: Acetonitrile (1.0% Acetic acid) B: Water (1.0% Acetic acid)			A: Acetonitrile B: Water		
Gradient Condition	Time (min)	A (%)	B (%)	Time (min	A (%)	B (%)	Time (min)	A (%)	B (%)	Time (min)	A (%)	B (%)
	0	70	30	0	20	80	0	22	78	0	30	70
	30	70	30	40	50	50	35	22	78	30	36	64
	31	100	0	45	100	0	36	100	0	50	39	61
	40	100	0	50	100	0	45	100	0	55	100	0
	41	70	30	51	20	80	46	22	78	60	100	0
	50	70	30	60	20	80	60	22	78	61	30	70
										70	30	70

HPLC, high-performance liquid chromatography.

**Table 2:** Detection wavelength, working range, regression equation, r^2^, detection limit (DL), and quantitation limit (QL) for each compound.

Analyte	Detection Wavelength (nm)	Working Range (µg/mL)	Regression Equation	r^2^	DL (µg/mL)	QL (µg/mL)
Glycyrrhizic acid	254	15.625–500	y=7650.5x−512.89	0.9990	5.24	15.89
Ginsenoside Rb_1_	195	50–800	y=4217.8x−7960.6	0.9996	4.40	13.33
Baicalin	277	15.625–1000	y=31,296x−44,462	0.9995	3.08	9.34
Saikosaponin A	203	7.8125–250	y=5809.3x−5866.6	0.9994	0.73	2.21
Saikosaponin B2	253	15.625–1000	y=20,863x+76,320	0.9990	3.46	10.49

**Table 3:** Content of five compounds in SSHT and the criteria established by Korean Herbal Pharmacopoeia (KHP).

Compound	Content in SSHT Dry Extract (mg/g)	Criteria in KHP (mg/g)
Glycyrrhizic acid	5.333 ± 0.040	1.6
Ginsenoside Rb_1_	1.301 ± 0.037	N/A *
Baicalin	55.148 ± 0.138	11.9
Saikosaponin A	0.216 ± 0.013	N/A
Saikosaponin B2	0.750 ± 0.007	N/A

* N/A: Not applicable, the content criteria are not specified in KHP.

## Data Availability

The datasets generated during and/or analyzed during the current study are available from the corresponding author upon reasonable request.

## Supplementary Materials

The following supporting information can be downloaded at: https://www.mdpi.com/article/10.3390/biom14040451/s1, File S1: Original blot images.
