# Supplementary material for: So Shiho Tang Reduces Inflammation in Lipopolysaccharide-Induced RAW 264.7 Macrophages and Dextran Sodium Sulfate-Induced Colitis Mice
Source: Biomolecules. 2024 Apr 7;14(4):451. doi: 10.3390/biom14040451 (PMC11047977; doi:10.3390/biom14040451)
Supplement: Supplementary file 1 [file biomolecules-14-00451-s001.zip › biomolecules-2916429 supplementary/biomolecules-2916429-original western blot of Figure3-5 .pdf]

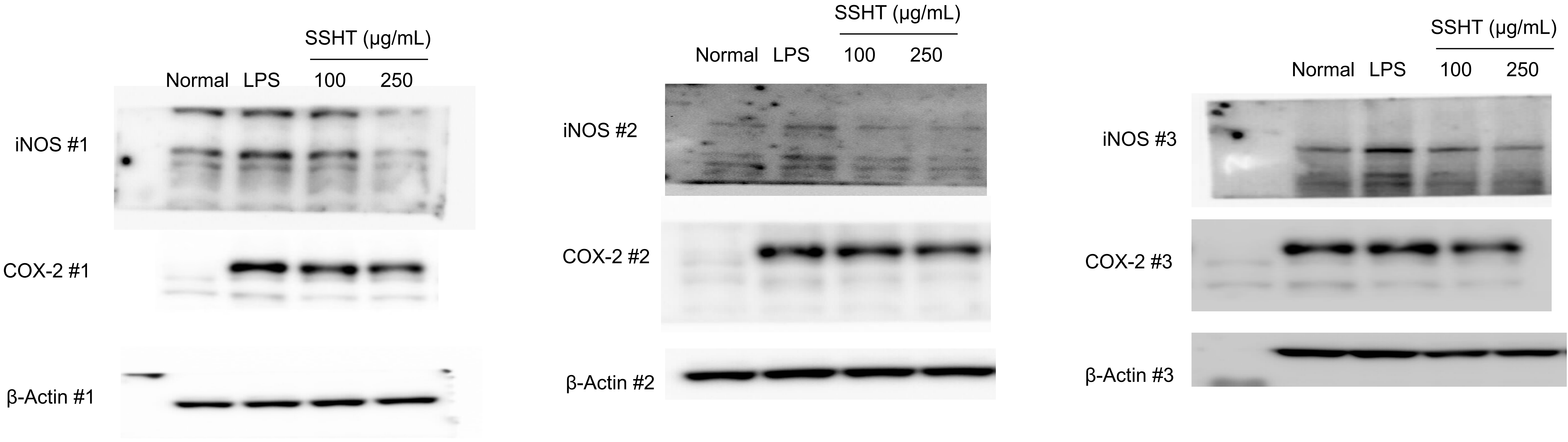

Figure 3. Effect of SSHT on nitrite production and inducible nitric oxide synthase (iNOS) and cyclooxygenase-2 (COX-2) abundance in LPS-stimulated RAW 264.7 cells.

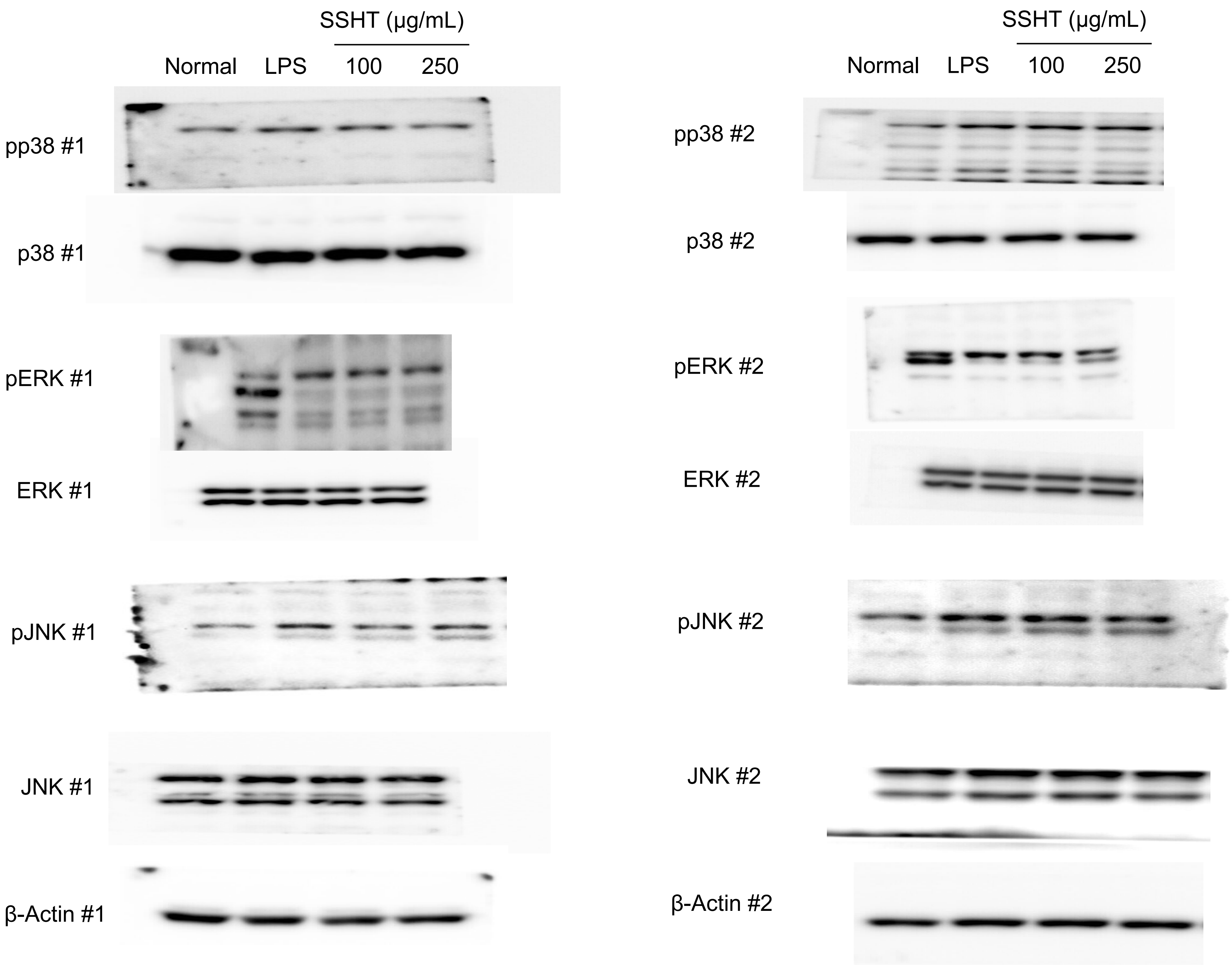

Figure 4. Effect of SSHT on p38, JNK, and ERK phosphorylation and total protein levels in LPS-stimulated RAW 264.7 cells.

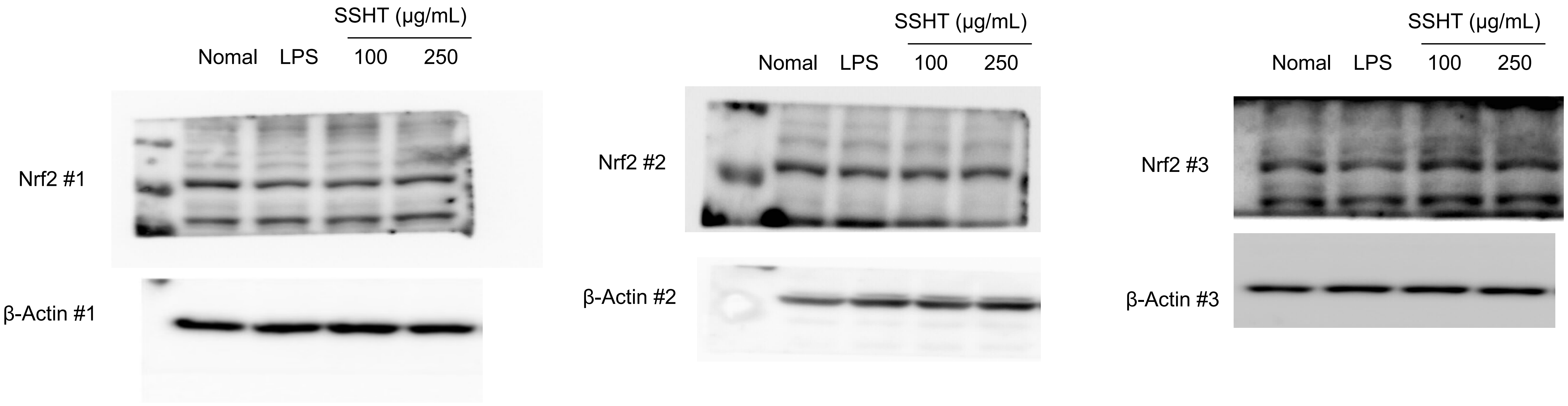

Figure 5. Effect of SSHT on nuclear factor erythroid 2-related factor 2 (NRF2) protein expression in LPS-stimulated RAW 264.7 cells.
