# Supplementary material for: So Shiho Tang Reduces Inflammation in Lipopolysaccharide-Induced RAW 264.7 Macrophages and Dextran Sodium Sulfate-Induced Colitis Mice
Source: Biomolecules. 2024 Apr 7;14(4):451. doi: 10.3390/biom14040451 (PMC11047977; doi:10.3390/biom14040451)
Supplement: Supplementary file 1 [file biomolecules-14-00451-s001.zip › biomolecules-2916429 supplementary/biomolecules-2916429-supplementary Table S1-S5.docx]

Journal: *Molecular and Cellular Biochemistry*

So Shiho Tang Reduces Inflammation in LPS-induced Raw264.7 Macrophages and DSS-induced Colitis Mice

Mei Tong He^1,#^, Geonha Park^2,#^, Do Hwi Park^1^, Minsik Choi^3^, Sejin Ku^3^, Seung Hyun Go^3^, Yun Gyo Lee^4^, Seok Jun Song^4^, Chang-Wook Ahn^5^, Young Pyo Jang^2, 3, 6,*^, Ki Sung Kang^1,*^

^1^College of Korean Medicine, Gachon University, Seongnam 13120, Republic of Korea; ellenho@gachon.ac.kr (M.T.H.), parkdo@gachon.ac.kr (D.H.P.), kkang@gachon.ac.kr (K.S.K.),

^2^Department of Life and Nanopharmaceutical Sciences, Graduate School, Kyung Hee University, Seoul 02447, Republic of Korea; ginapark0326@khu.ac.kr (G.P.)

^3^Department of Biomedical and Pharmaceutical Sciences, Graduate School, Kyung Hee University, Seoul 02447, Republic of Korea; alstlr7595@naver.com (M.C.), zbxl0910@khu.ac.kr (S.K.), 2021310688@khu.ac.kr (S.-H.G.), kyo3733@naver.com (Y.-G.L.), thd105@naver.com (S.-J.S.)

^4^Dr. Ahn’s Surgery Clinic, Osan 18144, Republic of Korea; acwe7@naver.com

^5^Department of Integrated Drug Development and Natural Products, Graduate School, Kyung Hee University, Seoul 02447, Republic of Korea; ypjang@khu.ac.kr (Y.-P.J.)

^#^These 2 authors contributed equally to this work.

^*^Correspondence: kkang@gachon.ac.kr (K.S.K.); Tel.: +82-31-750-5402 (K.S.K.); ypjang@khu.ac.kr (Y.-P.J.); Tel.: +82-2-961-9421 (Y.-P.J.)

Table S1. Validation results of method for Glycyrrhizic acid assay

| **System Suitability – Glycyrrhizic acid peak in sample solution** | | | | | | | | | | | | | |
| --- | --- | --- | --- | --- | --- | --- | --- | --- | --- | --- | --- | --- | --- |
| Capacity Factor  (k, retention factor) | | | Symmetry Factor  (S, tailing factor) | | | | Resolution  (Rs) | | Theoretical Plates number (N) | | | %RSD  (n$=$6) | |
| 7.08 | | | 1.03 | | | | 1.38 | | >2000 | | | 0.35 | |
| **Calibration curve data in quantitative assay** | | | | | | | | | | | | | |
| Analyte | | | | Regression equation | | | | R^2^ | | | Linear range (µg/mL) | | |
| Glycyrrhizic acid | | | | $y=7650.5x-512.89$ | | | | 0.9990 | | | 15.625 ~ 500 | | |
| **Detection Limit (DL) and Quantitation Limit (QL)** | | | | | | | | | | | | | |
| Analyte | | | Slope (S) | | | | Residual standard deviation (σ) | | DL (µg/mL) | | | QL (µg/mL) | |
| Glycyrrhizic acid | | | 7650.50 | | | | 12159.50 | | 5.24 | | | 15.89 | |
| **Precision – repeatability** | | | | | | | | | | | | | |
| 1. Three different concentration (n$=$3, area) | | | | | | | | 1. 100% concentration (n$=$6, area) | | | | | |
|  | 20 mg/mL | | | 30 mg/mL | | 40 mg/mL | |  | | Produced on Jul 1^st^ 2021 | | | Produced on Jul 12^th^ 2021 |
| Average | 312365.33 | | | 459322.00 | | 586960.67 | | Average | | 453752.50 | | | 445546.33 |
| SD | 829.54 | | | 2295.58 | | 4130.08 | | SD | | 6436.93 | | | 1546.67 |
| %RSD | 0.27 | | | 0.50 | | 0.70 | | %RSD | | 1.42 | | | 0.35 |
|  | | | | | | | | Total Average | | 449649.00 | | | |
|  | | | | | | | | Total SD | | 6187.63 | | | |
|  | | | | | | | | Total %RSD | | 1.38 | | | |
| **Precision – intermediate precision and Reproducibility** | | | | | | | | | | | | | |
|  | | | Intermediate precision (area) | | | | | | Reproducibility (mg/dose) | | | | |
|  | | | Produced on Jul 12^th^ 2021 | | | | | | Laboratory 1 | | | Laboratory 2 | |
|  | | | Analyzed on  Jul 14^th^ 2021 | | | | Analyzed on  Jul 16^th^ 2021 | |  |  |  |  |  |
| Average | | | 445546.33 | | | | 438166.50 | | 5.79 | | | 6.01 | |
| SD | | | 1546.67 | | | | 7407.47 | | 0.02 | | | 0.02 | |
| %RSD | | | 0.35 | | | | 1.69 | | 0.35 | | | 0.35 | |
| Total Average | | | 441856.40 | | | | | | 5.90 | | | | |
| Total SD | | | 6393.9 | | | | | | 0.12 | | | | |
| Total %RSD | | | 1.45 | | | | | | 2.03 | | | | |
| **Accuracy (average of n**$\mathbf{=}$**3, total n**$\mathbf{=}$**9)** | | | | | | | | | | | | | |
| Standard solution (µg/mL) | | | Measured Area | | | | Theory area | | %Recovery | | | %RSD | |
| 15.625 | | | 269480.33 | | | | 280742.83 | | 95.99 | | | 0.25 | |
| 62.5 | | | 445030.67 | | | | 450543.33 | | 98.78 | | | 1.85 | |
| 500 | | | 2171366.67 | | | | 2149219.17 | | 101.03 | | | 0.31 | |
| Total average of %Recovery (n$=$9) | | | | | | | | | 98.60 | | | | |
| Total SD | | | | | | | | | 2.38 | | | | |
| Total %RSD | | | | | | | | | 2.42 | | | | |
| The range of 95% confidence interval (%) | | | | | | | | | 96.77 ~ 100.43 | | | | |
| **Robustness (3 different columns)** | | | | | | | | | | | | | |
| Column | | *Area | | | %RSD  (n$=$3) | | | Capacity Factor (k) | | Symmetry Factor (S) | | | Resolution  (Rs) |
| ^a^Gemini | | 445546.33 | | | 0.35 | | | 7.08 | | 1.03 | | | 1.38 |
| ^b^Atlantis | | 438698.00 | | | 0.70 | | | 6.40 | | 1.06 | | | >2 |
| ^c^YMC | | 436507.00 | | | 0.29 | | | 5.60 | | 1.05 | | | 1.48 |
| ^a^ Phenomenex Gemini® C18 5 µm 4.6$\times$250 nm column; used for validation  ^b^ Waters Atlantis™ T3 5 µm LC column (4.6$\times$250 nm)  ^c^ Phenomenex LC column (4.6$\times$250 nm)  * Average of 3 injection of the sample solution; produced on Jul 12^th^ 2021 (30 mg/mL) | | | | | | | | | | | | | |

Table S2. Validation results of method for Ginsenoside Rb_1_ assay

| **System Suitability – Ginsenoside Rb_1_ peak in sample solution** | | | | | | | | | | | | | |
| --- | --- | --- | --- | --- | --- | --- | --- | --- | --- | --- | --- | --- | --- |
| Capacity Factor  (k, retention factor) | | | Symmetry Factor  (S, tailing factor) | | | | Resolution  (Rs) | | Theoretical Plates number (N) | | | %RSD  (n$=$6) | |
| 10.54 | | | 0.98 | | | | 2.64 | | >2000 | | | 0.17 | |
| **Calibration curve data in quantitative assay** | | | | | | | | | | | | | |
| Analyte | | | | Regression equation | | | | R^2^ | | | Linear range (µg/mL) | | |
| Ginsenoside Rb_1_ | | | | $y=4217.8x-7960.6$ | | | | 0.9996 | | | 50 ~ 800 | | |
| **Detection Limit (DL) and Quantitation Limit (QL)** | | | | | | | | | | | | | |
| Analyte | | | Slope (S) | | | | Residual standard deviation (σ) | | DL (µg/mL) | | | QL (µg/mL) | |
| Ginsenoside Rb_1_ | | | 4217.80 | | | | 5622.08 | | 4.40 | | | 13.33 | |
| **Precision – repeatability** | | | | | | | | | | | | | |
| 1. Three different concentration (n$=$3, area) | | | | | | | | 1. 100% concentration (n$=$6, area) | | | | | |
|  | 40 mg/mL | | | 60 mg/mL | | 80 mg/mL | |  | | Produced on Jul 15^th^ 2021 | | | Produced on Jul 16^th^ 2021 |
| Average | 738243.33 | | | 1103687.67 | | 1342199.67 | | Average | | 1101396.17 | | | 110212.33 |
| SD | 3226.79 | | | 20076.77 | | 8712.02 | | SD | | 6996.02 | | | 18174.95 |
| %RSD | 0.44 | | | 1.82 | | 0.65 | | %RSD | | 0.64 | | | 1.64 |
|  | | | | | | | | Total Average | | 1105304.25 | | | |
|  | | | | | | | | Total SD | | 28919.51 | | | |
|  | | | | | | | | Total %RSD | | 2.62 | | | |
| **Precision – intermediate precision and Reproducibility** | | | | | | | | | | | | | |
|  | | | Intermediate precision (area) | | | | | | Reproducibility (mg/dose) | | | | |
|  | | | Produced on Jul 15^th^ 2021 | | | | | | Laboratory 1 | | | Laboratory 2 | |
|  | | | Analyzed on  Jul 15^th^ 2021 | | | | Analyzed on  Jul 16^th^ 2021 | |  |  |  |  |  |
| Average | | | 1101396.17 | | | | 1059872.17 | | 1.233 | | | 1.144 | |
| SD | | | 6996.02 | | | | 17764.03 | | 0.008 | | | 0.004 | |
| %RSD | | | 0.64 | | | | 1.68 | | 0.674 | | | 0.311 | |
| Total Average | | | 1080634.17 | | | | | | 1.188 | | | | |
| Total SD | | | 25217.69 | | | | | | 0.05 | | | | |
| Total %RSD | | | 2.33 | | | | | | 3.92 | | | | |
| **Accuracy (average of n**$\mathbf{=}$**3, total n**$\mathbf{=}$**9)** | | | | | | | | | | | | | |
| Standard solution (µg/mL) | | | Measured Area | | | | Theory area | | %Recovery | | | %RSD | |
| 50 | | | 623565.00 | | | | 652522.58 | | 95.56 | | | 0.47 | |
| 200 | | | 943281.00 | | | | 974851.25 | | 96.76 | | | 0.31 | |
| 800 | | | 2212543.67 | | | | 2212358.08 | | 100.01 | | | 0.69 | |
| Total average of %Recovery (n$=$9) | | | | | | | | | 97.44 | | | | |
| Total SD | | | | | | | | | 2.04 | | | | |
| Total %RSD | | | | | | | | | 2.09 | | | | |
| The range of 95% confidence interval (%) | | | | | | | | | 95.88 ~ 99.01 | | | | |
| **Robustness (3 different columns)** | | | | | | | | | | | | | |
| Column | | *Area | | | %RSD  (n$=$3) | | | Capacity Factor (k) | | Symmetry Factor (S) | | | Resolution  (Rs) |
| ^a^YMC | |  | | |  | | |  | |  | | |  |
| ^b^Atlantis | | 1091055.67 | | | 0.69 | | | 10.88 | | 0.98 | | | >2 |
| ^c^Gemini | | 1094251.00 | | | 0.32 | | | 10.41 | | 0.91 | | | >2 |
| ^a^ YMC-Pack Pro C18 LC column 4.6$\times$150 nm column (I.D. S-5 µm,12 nm); used for validation  ^b^ Waters Atlantis™ T3 5 µm LC column (4.6$\times$150 nm)  ^c^ Phenomenex Gemini® C18 5 µm 4.6$\times$150 nm column  * Average of 3 injection of the sample solution; produced on July 15^th^ 2021 (60 mg/mL) | | | | | | | | | | | | | |

Table S3. Validation results of method for Baicalin assay

| **System Suitability – Baicalin peak in sample solution** | | | | | | | | | | | | | |
| --- | --- | --- | --- | --- | --- | --- | --- | --- | --- | --- | --- | --- | --- |
| Capacity Factor  (k, retention factor) | | | Symmetry Factor  (S, tailing factor) | | | | Resolution  (Rs) | | Theoretical Plates number (N) | | | %RSD  (n$=$6) | |
| 10.33 | | | 0.98 | | | | 2.16 | | >2000 | | | 0.20 | |
| **Calibration curve data in quantitative assay** | | | | | | | | | | | | | |
| Analyte | | | | Regression equation | | | | R^2^ | | | Linear range (µg/mL) | | |
| Baicalin | | | | $y=31296x-44462$ | | | | 0.9995 | | | 15.625 ~ 1000 | | |
| **Detection Limit (DL) and Quantitation Limit (QL)** | | | | | | | | | | | | | |
| Analyte | | | Slope (S) | | | | Residual standard deviation (σ) | | DL (µg/mL) | | | QL (µg/mL) | |
| Baicalin | | | 31296 | | | | 29225.5 | | 3.08 | | | 9.34 | |
| **Precision – repeatability** | | | | | | | | | | | | | |
| 1. Three different concentration (n$=$3, area) | | | | | | | | 1. 100% concentration (n$=$6, area) | | | | | |
|  | 30 mg/mL | | | 40 mg/mL | | 50 mg/mL | |  | | Produced on Jul 7^th^ 2021 | | | Produced on Jul 8^th^ 2021 |
| Average | 7516008.00 | | | 10109664.33 | | 12441251.67 | | Average | | 10038559.67 | | | 10040051.50 |
| SD | 10550.80 | | | 140966.99 | | 11932.58 | | SD | | 119140.80 | | | 19728.60 |
| %RSD | 0.14 | | | 1.39 | | 0.10 | | %RSD | | 1.19 | | | 0.20 |
|  | | | | | | | | Total Average | | 10039305.58 | | | |
|  | | | | | | | | Total SD | | 81422.25 | | | |
|  | | | | | | | | Total %RSD | | 0.81 | | | |
| **Precision – intermediate precision and Reproducibility** | | | | | | | | | | | | | |
|  | | | Intermediate precision (area) | | | | | | Reproducibility (mg/dose) | | | | |
|  | | | Produced on Jul 8^th^ 2021 | | | | | | Laboratory 1 | | | Laboratory 2 | |
|  | | | Analyzed on  Jul 8^th^ 2021 | | | | Analyzed on  Jul 9^th^ 2021 | |  |  |  |  |  |
| Average | | | 10040051.50 | | | | 10147059.00 | | 31.58 | | | 31.44 | |
| SD | | | 19728.60 | | | | 19443.04 | | 0.06 | | | 0.04 | |
| %RSD | | | 0.20 | | | | 0.19 | | 0.20 | | | 0.11 | |
| Total Average | | | 10093555.25 | | | | | | 31.51 | | | | |
| Total SD | | | 58920.64 | | | | | | 0.09 | | | | |
| Total %RSD | | | 0.58 | | | | | | 0.27 | | | | |
| **Accuracy (average of n**$\mathbf{=}$**3, total n**$\mathbf{=}$**9)** | | | | | | | | | | | | | |
| Standard solution (µg/mL) | | | Measured Area | | | | Theory area | | %Recovery | | | %RSD | |
| 15.625 | | | 5395647.33 | | | | 5247725.58 | | 102.82 | | | 0.50 | |
| 125 | | | 7225051.67 | | | | 6948583.25 | | 103.98 | | | 0.19 | |
| 1000 | | | 20759403.00 | | | | 20761710.92 | | 99.99 | | | 0.38 | |
| Total average of %Recovery (n$=$9) | | | | | | | | | 102.26 | | | | |
| Total SD | | | | | | | | | 1.81 | | | | |
| Total %RSD | | | | | | | | | 1.77 | | | | |
| The range of 95% confidence interval (%) | | | | | | | | | 100.87 ~ 103.65 | | | | |
| **Robustness (3 different columns)** | | | | | | | | | | | | | |
| Column | | *Area | | | %RSD  (n$=$3) | | | Capacity Factor (k) | | Symmetry Factor (S) | | | Resolution  (Rs) |
| ^a^Luna | | 10036129.67 | | | 0.30 | | | 10.33 | | 0.99 | | | 2.16 |
| ^b^YMC | | 10298966.00 | | | 0.08 | | | 12.54 | | 1.12 | | | 1.82 |
| ^c^Atlantis | | 10111713.00 | | | 0.14 | | | 11.88 | | 1.06 | | | 1.42 |
| ^a^ Phenomenex Luna® C18(2) 100Å 5 µm 4.6$\times$250 nm column; used for validation  ^b^ YMC-Pack Pro C18 LC column 4.6$\times$250 nm column (I.D. S-5 µm,12 nm)  ^c^ Waters Atlantis™ T3 5 µm LC column (4.6$\times$250 nm)  * Average of 3 injection of the sample solution; produced on Jul 8^th^ 2021 (40 mg/mL) | | | | | | | | | | | | | |

Table S4. Validation results of method for Saikosaponin A assay

| **System Suitability – Saikosaponin A peak in sample solution** | | | | | | | | | | | | | |
| --- | --- | --- | --- | --- | --- | --- | --- | --- | --- | --- | --- | --- | --- |
| Capacity Factor  (k, retention factor) | | | Symmetry Factor  (S, tailing factor) | | | | Resolution  (Rs) | | Theoretical Plates number (N) | | | %RSD  (n$=$6) | |
| 17.49 | | | 1.11 | | | | >2 | | >2000 | | | 1.85 | |
| **Calibration curve data in quantitative assay** | | | | | | | | | | | | | |
| Analyte | | | | Regression equation | | | | R^2^ | | | Linear range (µg/mL) | | |
| Saikosaponin A | | | | $y=5809.3x-5866.6$ | | | | 0.9994 | | | 7.8125 ~ 250 | | |
| **Detection Limit (DL) and Quantitation Limit (QL)** | | | | | | | | | | | | | |
| Analyte | | | Slope (S) | | | | Residual standard deviation (σ) | | DL (µg/mL) | | | QL (µg/mL) | |
| Saikosaponin A | | | 5809.3 | | | | 1303.19 | | 0.73 | | | 2.21 | |
| **Precision – repeatability** | | | | | | | | | | | | | |
| 1. Three different concentration (n$=$3, area) | | | | | | | | 1. 100% concentration (n$=$6, area) | | | | | |
|  | 2500 mg/mL | | | 3000 mg/mL | | 3500 mg/mL | |  | | Produced on Aug 3^rd^ 2022 | | | Produced on Aug 5^th^ 2022 |
| Average | 238041.33 | | | 287454.33 | | 359640.33 | | Average | | 282958.50 | | | 289001.00 |
| SD | 4500.80 | | | 3659.95 | | 3084.93 | | SD | | 5234.96 | | | 3349.78 |
| %RSD | 1.89 | | | 1.27 | | 0.86 | | %RSD | | 1.85 | | | 1.16 |
|  | | | | | | | | Total Average | | 285979.75 | | | |
|  | | | | | | | | Total SD | | 5245.47 | | | |
|  | | | | | | | | Total %RSD | | 1.83 | | | |
| **Precision – intermediate precision and Reproducibility** | | | | | | | | | | | | | |
|  | | | Intermediate precision (area) | | | | | | Reproducibility (mg/dose) | | | | |
|  | | | Produced on Aug 3^rd^ 2022 | | | | | | Laboratory 1 | | | Laboratory 2 | |
|  | | | Analyzed on  Aug 3^rd^ 2022 | | | | Analyzed on  Aug 4^th^ 2021 | |  |  |  |  |  |
| Average | | | 282958.50 | | | | 278812.67 | | 0.095 | | | 0.092 | |
| SD | | | 5234.96 | | | | 2415.15 | | 0.002 | | | 0.001 | |
| %RSD | | | 1.85 | | | | 0.87 | | 1.889 | | | 1.409 | |
| Total Average | | | 280885.58 | | | | | | 0.094 | | | | |
| Total SD | | | 4449.23 | | | | | | 0.002 | | | | |
| Total %RSD | | | 1.58 | | | | | | 2.040 | | | | |
| **Accuracy (average of n**$\mathbf{=}$**3, total n**$\mathbf{=}$**9)** | | | | | | | | | | | | | |
| Standard solution (µg/mL) | | | Measured Area | | | | Theory area | | %Recovery | | | %RSD | |
| 7.8125 | | | 158980.33 | | | | 164902.75 | | 96.41 | | | 1.45 | |
| 62.5 | | | 324845.00 | | | | 331228.92 | | 98.07 | | | 1.45 | |
| 250 | | | 857056.33 | | | | 882675.92 | | 97.10 | | | 1.14 | |
| Total average of %Recovery (n$=$9) | | | | | | | | | 97.19 | | | | |
| Total SD | | | | | | | | | 1.38 | | | | |
| Total %RSD | | | | | | | | | 1.42 | | | | |
| The range of 95% confidence interval (%) | | | | | | | | | 96.13 ~ 98.25 | | | | |
| **Robustness (3 different columns)** | | | | | | | | | | | | | |
| Column | | *Area | | | %RSD  (n$=$3) | | | Capacity Factor (k) | | Symmetry Factor (S) | | | Resolution  (Rs) |
| ^a^Gemini | | 283306.00 | | | 0.13 | | | 17.50 | | 1.08 | | | >2 |
| ^b^Atlantis | | 223473.33 | | | 1.36 | | | 19.23 | | 0.92 | | | >2 |
| ^c^Luna | | 270423.00 | | | 1.65 | | | 17.59 | | 1.14 | | | >2 |
| ^a^ Phenomenex Gemini® C18 110Å 5 µm 4.6$\times$250 nm column; used for validation  ^b^ Waters Atlantis™ T3 5 µm LC column (4.6$\times$250 nm)  ^c^ Phenomenex Luna® C18(2) 100Å 5 µm 4.6$\times$250 nm column  * Average of 3 injection of the sample solution; produced on Aug 3^rd^ 2022 (3000 mg/mL) | | | | | | | | | | | | | |

Table S5. Validation results of method for Saikosaponin B2 assay

| **System Suitability – Saikosaponin B2 peak in sample solution** | | | | | | | | | | | | | |
| --- | --- | --- | --- | --- | --- | --- | --- | --- | --- | --- | --- | --- | --- |
| Capacity Factor  (k, retention factor) | | | Symmetry Factor  (S, tailing factor) | | | | Resolution  (Rs) | | Theoretical Plates number (N) | | | %RSD  (n$=$6) | |
| 17.15 | | | 0.99 | | | | >2 | | >2000 | | | 0.35 | |
| **Calibration curve data in quantitative assay** | | | | | | | | | | | | | |
| Analyte | | | | Regression equation | | | | R^2^ | | | Linear range (µg/mL) | | |
| Saikosaponin B2 | | | | $y=20863x+76320$ | | | | 0.9990 | | | 15.625 ~ 1000 | | |
| **Detection Limit (DL) and Quantitation Limit (QL)** | | | | | | | | | | | | | |
| Analyte | | | Slope (S) | | | | Residual standard deviation (σ) | | DL (µg/mL) | | | QL (µg/mL) | |
| Saikosaponin B2 | | | 20863 | | | | 21883.90 | | 3.46 | | | 10.49 | |
| **Precision – repeatability** | | | | | | | | | | | | | |
| 1. Three different concentration (n$=$3, area) | | | | | | | | 1. 100% concentration (n$=$6, area) | | | | | |
|  | 2500 mg/mL | | | 3000 mg/mL | | 3500 mg/mL | |  | | Produced on Aug 3^rd^ 2022 | | | Produced on Aug 5^th^ 2022 |
| Average | 760385.33 | | | 953845.00 | | 1244293.67 | | Average | | 995025.67 | | | 949374.67 |
| SD | 4881.68 | | | 1416.72 | | 14543.49 | | SD | | 3452.46 | | | 5339.35 |
| %RSD | 0.64 | | | 0.15 | | 1.17 | | %RSD | | 0.35 | | | 0.56 |
|  | | | | | | | | Total Average | | 989495.58 | | | |
|  | | | | | | | | Total SD | | 10570.33 | | | |
|  | | | | | | | | Total %RSD | | 1.07 | | | |
| **Precision – intermediate precision and Reproducibility** | | | | | | | | | | | | | |
|  | | | Intermediate precision (area) | | | | | | Reproducibility (mg/dose) | | | | |
|  | | | Produced on Aug 3^rd^ 2022 | | | | | | Laboratory 1 | | | Laboratory 2 | |
|  | | | Analyzed on  Aug 3^rd^ 2022 | | | | Analyzed on  Aug 4^th^ 2021 | |  |  |  |  |  |
| Average | | | 995025.67 | | | | 983965.50 | | 0.088 | | | 0.093 | |
| SD | | | 3452.46 | | | | 12668.64 | | 0.000 | | | 0.001 | |
| %RSD | | | 0.35 | | | | 1.29 | | 0.301 | | | 0.760 | |
| Total Average | | | 942200.17 | | | | | | 0.091 | | | | |
| Total SD | | | 24222.80 | | | | | | 0.003 | | | | |
| Total %RSD | | | 2.49 | | | | | | 3.020 | | | | |
| **Accuracy (average of n**$\mathbf{=}$**3, total n**$\mathbf{=}$**9)** | | | | | | | | | | | | | |
| Standard solution (µg/mL) | | | Measured Area | | | | Theory area | | %Recovery | | | %RSD | |
| 15.625 | | | 786998.33 | | | | 670352.83 | | 117.40 | | | 0.32 | |
| 62.5 | | | 1376733.00 | | | | 1180587.17 | | 116.61 | | | 0.96 | |
| 1000 | | | 13037524.33 | | | | 10939325.17 | | 119.18 | | | 0.84 | |
| Total average of %Recovery (n$=$9) | | | | | | | | | 117.73 | | | | |
| Total SD | | | | | | | | | 1.31 | | | | |
| Total %RSD | | | | | | | | | 1.12 | | | | |
| The range of 95% confidence interval (%) | | | | | | | | | 116.72 ~ 118.74 | | | | |
| **Robustness (3 different columns)** | | | | | | | | | | | | | |
| Column | | *Area | | | %RSD  (n$=$3) | | | Capacity Factor (k) | | Symmetry Factor (S) | | | Resolution  (Rs) |
| ^a^Gemini | | 992501.33 | | | 0.20 | | | 17.14 | | 1.00 | | | >2 |
| ^b^Atlantis | | 955171.67 | | | 1.12 | | | 19.70 | | 1.08 | | | >2 |
| ^c^Luna | | 945518.00 | | | 0.95 | | | 17.91 | | 0.93 | | | >2 |
| ^a^ Phenomenex Gemini® C18 110Å 5 µm 4.6$\times$250 nm column; used for validation  ^b^ Waters Atlantis™ T3 5 µm LC column (4.6$\times$250 nm)  ^c^ Phenomenex Luna® C18(2) 100Å 5 µm 4.6$\times$250 nm column  * Average of 3 injection of the sample solution; produced on Aug 3^rd^ 2022 (3000 mg/mL) | | | | | | | | | | | | | |
